# Can Worried Parents Predict Effects of Video Games on Their Children? A Case-Control Study of Cognitive Abilities, Addiction Indicators and Wellbeing

**DOI:** 10.3389/fpsyg.2020.586699

**Published:** 2021-01-15

**Authors:** Andreas Lieberoth, Anne Fiskaali

**Affiliations:** ^1^Interacting Minds Centre, Aarhus University, Aarhus, Denmark; ^2^Danish School of Education, Aarhus University, Aarhus, Denmark; ^3^Department of Psychology and Behavioural Sciences, Aarhus BSS, Aarhus University, Aarhus, Denmark

**Keywords:** parenting, video games [psychology], video games addiction, cognitive abilities, wellbeing, screen time, executive control

## Abstract

Many parents worry over their children’s gaming habits, but to what extent do such worries match any detrimental effects of excessive gaming? We attempted to answer this question by comparing children of highly concerned parents with other adolescents of the same age. A cohort of parents who identified as highly concerned over their children’s video game habits were recruited for a public study in collaboration with a national television network. Using an online experimental platform in conjunction with surveys of parents’ beliefs and attitudes, we compared their children to age-matched peers in an exploratory case-control study. The scores of children with highly concerned parents on tests of cognitive control (cued task-switching and Iowa Gambling Task) and psychological wellbeing (WHO-5) were statistically similar to controls, suggesting no selective cognitive or psychological detriments from gaming or otherwise in the cases with concerned parents. The case group, however, did spend more time gaming, and scored higher than controls on problem gaming indicators (Gaming Addiction Scale), which also correlated negatively with wellbeing. Within the case group, wellbeing effects seemed mainly to consist in issues of relaxation and sleep, and related to gaming addiction indicators of playing to forget real-world problems, and the feeling of neglecting non-gaming activities. Where most results of research staged for TV never get published, making it difficult to interpret both methods and results, this paper describes findings and participant recruitment in detail. The relationship between parental concern and children’s gaming is discussed, as is the merits and challenges of research conducted with media, such as TV programs and their recruited on-screen participants.

## Introduction

Many parents worry about the time their children spend on video games, and debates concerning the impact of video games on, e.g., mental wellbeing, behavior, and cognitive functioning have become stables in societal conversations. Parents want their children to have good lives—and regulating early adolescent behavior can be challenging. This creates dilemmas for parents to heavy gamers. While worry has been found to correlate with restrictive practices and negatively with supportive behaviors (Lieberoth and Lundsgaard, 2020), autonomy supporting parenting approaches seem to enhance acceptance of rules and reduce media use among teens (Padilla-Walker et al., 2019; Weinstein and Przybylski, 2019). Yet parents struggle to judge appropriate levels of “screen time,” and popular concern augmented through media is a driver of policy preference and even research priorities in mental health and substance abuse domains (Hallam, 2002; Slater et al., 2009). Since the stakes of scientific evidence in this arena are so high, and the implications far reaching, close scrutiny of the data and interpretations is therefore crucial (Choudhury and Mckinney, 2013, p. 200). As such, a focus on the practices and beliefs of parent should be a focus for scientists and councilors alongside the potential psychological, developmental, or social effects of emerging digital media uses. This study therefore set out to investigate whether children of highly concerned parents actually displayed signs of problems related to video games including wellbeing, cognitive detriments, and the various symptoms of clinical addiction used in common diagnostic questionnaires compared with other adolescents in the same age group.

In early summer of 2018, we were approached by a television journalist, who based on a BBC program where the brains of young gamers were examined using electroencephalography (EEG), wanted to know if we could do a similar study for Danish TV2 in our lab. On further inspection, we were unable to find research supporting the technique used by the private contractor appearing on The Victoria Derbyshire program (BBC, 2018) to demonstrate that a small on-screen sample of children’s brains suffered detriments from their heavy video game play. We were skeptical of the fact that no cognitive testing had been conducted in conjunction with the electroencephalographical measures for criterion validity, that the sample was too small to render statistically meaningful findings, and that the contractor used no control group to validate the notion that any of their observations should be related specifically to the gaming habits of the young subjects. In the resulting discussion with TV2, we pointed to the tendency to, with notable exceptions (e.g., Owen et al., 2010), use few people as human interest cases in television programming, taking correlation for causality, and the need for larger samples to accurately assess statistical effects of games on the players in question. We were also hesitant about using neuroimaging to illustrate differences between children who game a lot and those who do not. Instead, we converged on a series of cognitive tests combined with a commonly used gaming addiction self-report scale. The producer was positive that she would be able to do better than the BBC piece and recruit a large number of concerned parents for our re-imagined study. Since TV pieces often revolve around concerned parents and their children, we agreed that it would be interesting to base our hypotheses on parental concern. We thus decided to ask: Are parental concerns a good predictor of negative effects of gaming? Do worried parents’ children differ from other kids their age in executive functioning, downregulation of appealing but ultimately detrimental choices, or general wellbeing? Issues that commonly appear in the public debate (Størup and Lieberoth, 2020), and worry some parents quite a bit (Lieberoth and Lundsgaard, 2020).

This comparative study first and foremost investigates whether *parents’ concern* about their children’s (age 12–17) video game play is an accurate predictor of issues related to healthy cognitive and psychological functioning.

As a second objective, the study looks for *crossectional predictors* of issues related to healthy cognitive and psychological functioning, within data from the *children* alone.

Finally, the study is a *broad exploration* of parental concerns, looking for *predictors of worry*, as well as describing the items of two measures commonly used to map children’s experiences of gaming problems and wellbeing [Game Addiction Scale (GAS) and WHO5].

### Understanding Parental Concern

New technologies always come with challenges that cannot easily be solved using existing frames of interpretation and intervention (Zuboff, 2019). While concerns over screen time in general and video games in particular sometimes address media quality (Soper and Miller, 1983) or objectionable content (Kuipers, 2006), concern discourses prominently feature mental and physical health and cognitive development (Størup and Lieberoth, 2020), variously understood as direct effect of exposure to media technologies or as displacement of more worthwhile pursuits (Choudhury and Mckinney, 2013; Przybylski and Weinstein, 2017; Lieberoth and Lundsgaard, 2020). While some studies find that effects of game time are small or at least very complex (Yang et al., 2013; Przybylski and Weinstein, 2017; Ferguson and Wang, 2019; Jensen et al., 2019), others have identified quadratic relationships between game time and mental wellbeing, suggesting that any dramatic impact should be found at the very high end of daily/weekly media use (Przybylski et al., 2019). As such, there may be an important distinction between general populations with moderate use patterns and more extreme cases. Indeed, cases of physiological conditions and discomforts have been reported in high- but rarely low-involvement eSports-players (Zwibel et al., 2019), and negative relationships with prosocial behavior have been found not for gaming in general, but for people involved in high-frequency competitive gaming (Lobel et al., 2017). Such emerging patterns suggest that if contemporary youth gaming cultures have detrimental effects beyond generational conflicts and time diverted from other activities, they might be found at the very high end of gaming behavior, rather than in the broad middle of social and casual play.

In this climate, then, it can be difficult for parents to avid gamers to judge whether their children’s play behavior warrants concern, and to which extent the time spent has a detrimental impact on cognitive development and psychological wellbeing. As such, this study focused on children of parents who subjectively believed that their children were at the problematic end of the gaming spectrum and were motivated enough by their distresses to enroll themselves and their adolescent in a study about problematic gaming.

### Cognitive Effects

Parental worry about games often concerns children’s cognitive functioning, popularly phrased as effects “on the brain” (Størup and Lieberoth, 2020). The causal pathways by which frequency of gaming has been proposed to act as a factor in individuals’ health status is extremely broad, “from the amount of time spent on these activities, from the neglect of other activities and priorities, from risky behaviors associated with gaming or its context, from the adverse consequences of gaming, or from the combination” (The World Health Organization, 2018b). To match this, an expanding body of research has found variations of fluid, often play- and game-dependent (Dale et al., 2019) associations between heavy gaming and cognitive processes ranging from perception to cognitive control, reward processing, and decision making (Bailey et al., 2010, 2011). Relationships between gaming and cognitive function have been tested using traditional measures of cognitive flexibility and control such as task-switching, Stroop, and N-back tasks (Colzato et al., 2013; Dong et al., 2014; Cardoso-Leite et al., 2016), which measure participants’ ability to cope with multiple task demands requiring prolonged concentration and attentiveness to specific instructions (Logan and Gordon, 2001). Studies focused on frequent versus non-frequent players have found gamers, or subjects trained using games, to perform better on cognitive tasks, especially related to visual attention and response execution (Anguera et al., 2013; Greitemeyer, 2019), but sometimes poorer in terms of response inhibition (Colzato et al., 2013; Steenbergen et al., 2015).

Directionality has also been discussed. Addictive behaviors have been linked to impaired regulatory control in favor of rewarding behaviors in, e.g., cannabis dependence, and some studies have identified neural correlates that may underlie such dysregulation (Ma et al., 2010; Zhou et al., 2018). Based on such research, some neurobiological studies have also worked to identify issues with the structural connectivity underlying cognitive control in Internet or gaming addiction (Ma et al., 2010; Dong et al., 2014; Wang et al., 2018). In studies focusing on individuals labeled addicts, findings tend to point to impaired cognitive control (Ma et al., 2010; Dong et al., 2014; Wang et al., 2018), suggesting that certain individuals may be more at risk of developing uncontrollable gaming behaviors. This matches a recent longitudinal study of child development which, despite concluding no relationship between gaming in late childhood and later DSM symptoms, found that children with ADHD symptoms were more likely to increase rather than decrease their gaming with age (Stenseng et al., 2019).

Yet other studies have looked for more proximate effects, finding that exposure to games perceived as difficult reduced cognitive control following play (Engelhardt et al., 2015) and that relations between play behavior and adolescent adjustment is quite complex (Verheijen et al., 2019). This emerging literature suggests that when discussing cognitive outcomes, individual differences, short- versus long-term effects, and gaming uses and gratifications in response to life situations are worth considering when interpreting parents’ worries about children’s gaming, including when they observe behaviors like aggression toward other players (McInroy and Mishna, 2017) or stress and short temperedness (Bavelier et al., 2011; Ferguson et al., 2016).

### Wellbeing

Numerous studies have examined the relationship between media use and wellbeing, variously framed in terms of happiness, general welfare, psychological distress, and psychiatric symptoms. While online video games sometimes have positive outcomes in terms of learning and social connectedness (Hanghøj et al., 2018) or on wellbeing (Pallavicini et al., 2018), the research literature also suggests correlations to depressive symptoms, suicidal ideations, alienation, eating disorders, and academic difficulties (for a review, see Strasburger et al., 2010). For games, the association with mental wellbeing seems especially prevalent for very frequent, or very infrequent, participants in the youth gaming culture (Przybylski and Weinstein, 2017). Indeed, associations between gaming and wellbeing have been found to be related to perceived social support (Sarriera et al., 2012; Kaczmarek and Dra̧żkowski, 2014), need frustration on- and offline (Allen and Anderson, 2018), neighborhood circumstances (Kim and Ahn, 2016), and escapism motives (Kaczmarek and Dra̧żkowski, 2014). Social alienation has been found related to the gratifications reported for violent games (Slater, 2003), but social support through player communities has also been found to be a psychological resource for gamers (Kaczmarek and Dra̧żkowski, 2014). This complexity matches overall findings that statistical relationships between digital media use and psychosocial wellbeing are statistically small, non-monotonic and shifting over time (Yang et al., 2013; Przybylski and Weinstein, 2017; Ferguson and Wang, 2019; Jensen et al., 2019), and subject to complex mediation relationships (Rasmussen et al., 2020). Indeed, factors like cyberbullying, sleep, and physical exercise have been found to attenuate negative relationships between digital media use and wellbeing, suggesting indirect causal pathways (Viner et al., 2019). As with dysfunctional behavior in other domains, indicators of gaming addiction are also related to life satisfaction, loneliness, anxiety, depression, and academic performance (Sarda et al., 2016) raising questions of directionality in excessive gaming. That recent longitudinal research found small or no effects over time (Jensen et al., 2019; Coyne et al., 2020) and also suggests that momentary dysfunctional relationships between wellbeing and media use can be a passing state in young people’s development. As such, it seems crucial to understand the relationship between media practices and various indicators of wellbeing in the context of their broader psychological and social circumstances—online, in school, and at home.

### Addiction

Early research using the term “addiction” in the context of video games considered implications for families (Ishigaki, 1986) and how student councilors should address this “junk time” issue (Soper and Miller, 1983). While referring to excessive gaming as an addiction is not new, the debate over the legitimacy of a potential diagnosis has, however, recently intensified. The American Psychiatric Association (APA) has declined to recognize gaming addiction as a distinct diagnosis (2013), whereas WHO is planning to add both “gaming disorder” and “hazardous gaming” to the next revision of the International Classification of Disease (The World Health Organization, 2018a,b). Commentators have criticized the ICD addition on grounds of weak empirical and theoretical support, stressing that games are among many behaviors and technologies that engage people for prolonged periods of time (Dullur and Starcevic, 2017; Van Rooij et al., 2018), while others have encouraged the step to formalize a diagnosis in an effort to help those who are experiencing problems (Király and Demetrovics, 2017; van den Brink, 2017). In this debate, delineation (Billieux et al., 2017; Saunders et al., 2017) and differentiation from non-pathological behaviors (Kardefelt-Winther et al., 2017; Reed et al., 2019) has therefore become a central issue, to mitigate the risk of parents or health professionals overinterpreting individual cases of recreational gaming as psychological or behavioral pathology.

Leading into this debate, a myriad of surveys and screening tools for video gaming addiction have been developed (King et al., 2013; Kuss, 2013), all employing varying conceptualizations of the supposed condition, one notably being behavioral addiction (Kuss and Griffiths, 2012). In this framework, any behavior perceived as rewarding by the individual may escalate to the point of pathology (Griffiths, 2005), and sufferers may experience symptoms much resembling addiction to psychoactive substances, such as withdrawal and relapse when prompted to cease the perceived problematic behavior. In the case of gaming, an addicted individual would thus be heavily preoccupied with gaming, experience intrapersonal and interpersonal conflicts due to engagement in the activity, and be unable to quit gaming altogether. One example of such instruments is the short-form GAS for adolescents (Lemmens et al., 2009), which has been used in numerous research studies probing the effects of games on young people (Collins and Freeman, 2013; Irvine et al., 2013; Scharkow et al., 2014; Andreassen et al., 2016). While GAS scores have been found to be low in broad gamer populations (as per Scharkow et al., 2014), changes in GAS scores over time have been found related to cognitive tests like the Iowa Gambling Task (Irvine et al., 2013) and to small changes in wellbeing (Scharkow et al., 2014).

## The Present Study

The present study analyzes data collected in collaboration with the TV2 Denmark news network for a documentary program on worried parents, and the effects of video games on adolescents. Data was collected from dyads of parents and children and intended to compare children and parents from three typical school classes to children of parents who volunteered themselves and their child for the study because they were worried about the effect of video games on their children. Based on the broad public discourse (Haddon and Stald, 2009; Størup and Lieberoth, 2020) and the research literature outlined above, we decided to test general health and wellbeing (WHO5), cognitive control (cued task switching), resistance to detrimental choices during decision making (Iowa gambling task), problematic gaming behavior (GAS), and the child’s game time per week (self-reported).

### Hypotheses and Exploratory Analyses

H1: If parental worry is warranted in children assigned to the “concern” group, we hypothesized that there should be a significantly different level (*lower cognitive tests and WHO5, higher GAS*) of those scores (see pre-registration).

H2: If time displacement is a central issue, we hypothesized that game time should be treated as a mediator for the other variables investigated in the study.

H3: Finally, it may be that parents are inadequately prepared to judge the relationship between gaming and other issues. As such, if there is a simple direct relationship between time spent playing and various issues, or a more complex relationship mediated by problematic gaming behavior, then children’s weekly game time may be a better predictor than parents’ level of concern.

Furthermore, correlational analyses will explore the scores on, and relationships between, other variables in the dataset. Using both Likert scales and written answers to open-ended questions, we explore what outcomes of gaming parents are most concerned about.

Given the high number of tests, all *p*-values are adjusted with false-discovery rate (FDR) correction where multiple comparisons occur in hypothesis testing.

## Materials and Methods

### Participants

Ninety-eight parents responded to media and online calls for “concerned parents.” Out of these, 67 case dyads completed the study along with 53 comparison dyads recruited through a local school. Children’s ages ranged from 12 to 17 (*M* = 13.09, *SD* = 1.16).

### Recruitment and Procedure

The study was approved by lab and regional IRBs prior to data collection.

Parent–child dyads were recruited together. The TV station ran TV spots and online invitations, supplemented with Twitter adds, in order to recruit a case group of parents who were highly concerned about the gaming habits of their child of 12–17. While three families were separately recruited to appear on-screen, the larger body only contributed their data and was aware that they would not appear on-screen. The participants received no monetary compensation but were offered a brief description of preliminary results comparing the case group with controls (i.e., no data for individual children). Three school classes and their parents were also recruited to participate in the study in conjunction with a lab visit at our university. School parents and children were blinded to the fact that they would primarily act as the control group but were given the general outline of the research questions.

Parents first filled in a separate questionnaire designed to map their concerns and rules and provide informed consent for their child’s participation. Their overall concern level was of our primary interest. Parents were encouraged to reach agreement on participation with their child before starting, and call him/her to the computer straight after they finished, in order to ensure completion. Parents and children were instructed not to look at each other’s answers or in other ways interfere. In order to connect children’s scores to parental concern and confirm parental approval, children entered an arbitrary code linking their response to that of their parent’s level of concern, supplied at the end of the parent’s questionnaire. The test session for children took on average 24 min, in which participants completed computerized versions of Cued Task Switching and Iowa Gambling tasks, inside a survey which include standardized versions of GAS and WHO5 as well as questions about media use and a set of more exploratory questions about participants’ own thinking about their media use and time spent gaming.

Depending on recruitment and convenience, some children filled in their test at a university lab, others at home. All parents filled in their surveys at home.

### Materials

#### Parent Survey

Parents were presented with written descriptions of procedure and eligibility before accessing a survey in the Qualtrics platform. The children’s portion of the study was conducted with the Linux-based PsyToolKit web platform (Stoet, 2010, 2016) to allow for a combination of survey questions and cognitive tests. Both contained detailed participant briefings and required informed consent.

Apart from background information, survey questions were either on a 6-point agree-disagree Likert scale or exploratory open-ended text/numbers, including hours and minutes for time use data.

Following background questions, parents were asked “how much of a problem do you, as a parent, think that your child’s gaming constitutes (in general)” on an 8-point expanded Likert scale including “extremely” agree/disagree options, and a “my child never games at all (as far as I know)” option.

We then asked a series of questions about common worries over video games, roughly divided into questions of time, wellbeing and cognitive effects inspired by concurrent work with popular media discourses (Størup and Lieberoth, 2020), and a series of questions about habits and rules in the home which are not analyzed here.

#### Children’s Survey

The children’s portion alternated between exploratory agree/disagree items, cognitive tests, and the GAS and WHO5 instruments.

The WHO5 instrument was used to measure wellbeing. It encapsulates aspects of everyday experience deemed crucial to everyday health and psychological functioning (Blom et al., 2012; Topp et al., 2015) with five agree-disagree statements concerning the past week.

The seven-item version of the GAS (Lemmens et al., 2009) was used to count gaming addiction symptoms. The frequency scale was adapted for six-point Likert scale responses. Each item addresses a criteria for gaming addiction. Lemmens et al. (2009) utilizes two cut-offs, in which an individual scoring either three or four (or higher) meets the relevant criteria. A cut-off of three was maintained in the revision of the scale for the current study due to its identical wording; in both the original and revised version scoring, three corresponds to “sometimes” experiencing a given symptom. Meeting four out of seven criteria would be considered addicted. GAS-7 was chosen for its focus on adolescents and that it despite its brevity has previously been found to adequately address diagnostic criteria for gaming addiction (King et al., 2013).

A cued switching task was used to measure cognitive control: in order to avoid interactions with the training of visuospatial cognitive processes from 3D action games (Bailey et al., 2011), a non-spatial and fairly boring repetitive task was chosen to allow lapses in concentration. CTS is a multitask response time procedure of the psychological refractory period variety, in which subjects make discrete responses to punctate stimuli that appear at controlled intervals (Welford, 1952; Logan and Gordon, 2001). In cued task switching, switching between a focus on the *shape* (square or circle?) and *color* (yellow or blue?) of each stimulus imposes ongoing switch costs (Meiran, 1996). Accuracy and response time for correct responses after switches from one focus to another are used as proxy measures of general cognitive control.

The Iowa Gambling Task (IGT) was used to measure resistance to attractive but detrimental choices (Bechara et al., 1994). Participants make ongoing choices from four virtual decks of cards, each revealing either gains or losses in a virtual currency. Two decks are advantageous on average, while two decks are disadvantageous but contain occasional attractive large gains. Because it is impossible to perform an exact mental calculation of net gains or losses per deck during play, broader “information sampling” is required (Irvine et al., 2013), and the subjects must therefore rely more on impulsive “gut feeling” (Damasio, 1994). Previous studies have found that subjects with impaired response inhibition and/or high sensitivity to immediate gratifications over long-term consequences, including those with high scores on problematic gambling or gaming (Bailey et al., 2013; Irvine et al., 2013; Trotzke et al., 2019) perform worse on this task than controls, because they show higher preference for the high reward but ultimately higher-punishment decks (here, decks 1 and 2).

### Data Analysis

Parent–child dyads were excluded if parent surveys were aborted before receiving codes for children’s survey (11 in both groups), or if children had not provided the parent code (11 in comparison group). Out of the participants who responded to the call for concerned parents and decided to participate after reading the study instructions, two also scored only 1 or 2 on their assessment that gaming was a problem and were eliminated from the dataset (as per the amended preregistration). Finally, eight children’s surveys were aborted at the first cognitive task and excluded, for a total of 67 valid parent–child responses (34 cases, 33 controls).

Non-parametric tests were used for group comparisons, as variances were unequal for a majority of the dependent variables. Data were analyzed using R/Jamovi (R Core Team, 2018; The Jamovi Project, 2019). Instead of ANCOVA analyses planned at the time of preregistration, mediation models were run using the Jamovi MedMod module. MedMod parametric bootstrapping was used when data was insufficient to calculate standard errors for mediation using the delta method.

One hundred percent of the children who volunteered for the case group were male versus only 36% in the comparison group. This difference was significant, χ^2^ = 412(2, 67), *p* < 0.001. Mean child age was 13.29 (*SD* = 1.59) for the case group and 12.88 (*SD* = 0.33) for the comparison group. The difference was not statistically significant.

**FIGURE 1:**
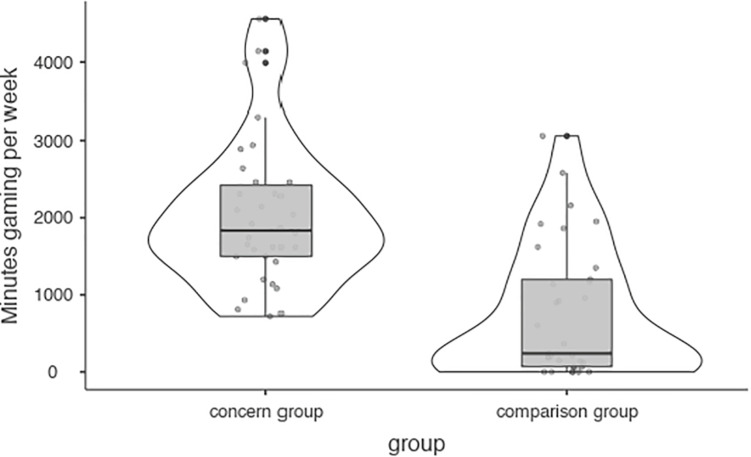
Minutes gaming per week.

Mean time spent on games reported is displayed in Table 1. The concern group spent significantly more minutes gaming per week than their peers *U* = 181.50 (*p* < 0.001, *d* = 1.39; 95% CI, 840–1,620) (Figure 1), with a larger difference reported for weekends, *U* = 149.00 (*p* < 0.001, *d* = 1.52; 95% CI, 180–330), than on weekdays *U* = 193.50 (*p* < 0.001, *d* = 1.13; 95% CI, 100–210).

**TABLE 1:** Minutes gaming.

	**Group**	***N***	**Mean**	**Median**	***SD***	***SE***
Per week	Concern	34	2,031.12	1,834	940.89	161.36
	Comparison	33	769.55	240	866.11	150.77
Weekdays	Concern	34	240.88	240	121.73	20.88
	Comparison	33	97.73	30.00	131.84	22.95
Weekend days	Concern	34	413.35	360.00	202.93	34.80
	Comparison	33	140.45	45.00	150.28	26.16

**FIGURE 2:**
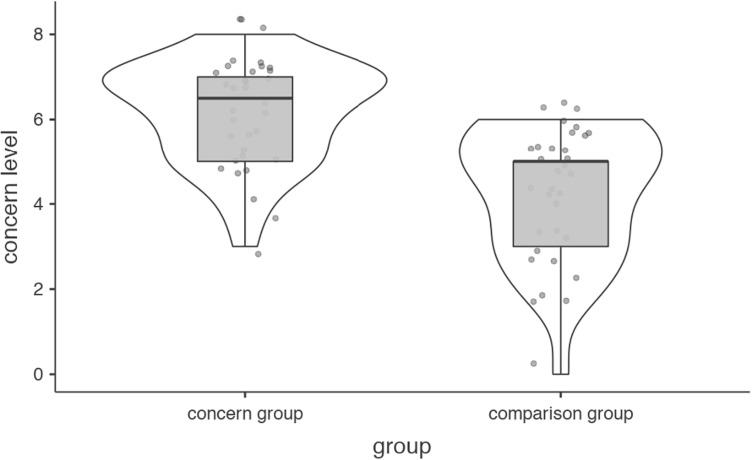
Parents’ concern level.

Mean self-rated parent concern score was 6.18 (*SD* = 1.22) for the case group and 4.21 (*SD* = 1.56) for the comparison group. The difference was large and significant *U* = 179.50 (*p* < 0.001, *d* = 1.41; 95% CI, 1–3) (Figure 2).

## Results

Results of non-parametric tests between group comparison testing included in the study hypotheses are displayed in Table 2 and values in Table 3. Detailed mediation analyses are supplied in the Supplementary Materials.

**TABLE 2:** Group comparisons on cognitive tests, general wellbeing and frequency of addiction related experiences.

	**Mann–Whitney *U***	***p***	***q***	**95% confidence interval**		**Cohen’s d**
				**Lower**	**Upper**	
Iowa gambling task	**527.00**	0.67	0.76	−12.00	7.00	−0.11
Cued task switching correct	**536.00**	0.76	0.76	−2.00	3.00	0.05
Repeat response time mean	334.00	0.01	0.01	−199.02	−44.15	−0.71
Response time mean	333.00	0.01	0.01	−237.37	−61.10	−0.74
WHO5	**509.50**	0.52	0.76	−0.20	0.60	0.16
Gaming Addiction Scale (GAS)	**253.00**	<0.001	0.01	0.43	1.00	1.04

**TABLE 3:** Group scores on cognitive tests, general wellbeing and frequency of addiction related experiences.

	**Group**	***N***	**Mean**	**Median**	***SD***	***SE***
Iowa gambling task detrimental deck picks	Concern group	34	46.35	50.50	19.86	3.41
	Comparison group	33	48.52	51.00	20.34	3.54
Cued task switching correct responses	Concern group	34	39.35	41.00	7.43	1.27
	Comparison group	33	39.00	41.00	8.05	1.40
CTS response time (repeat)	Concern group	34	640.70	634.68	179.96	30.86
	Comparison group	33	779.15	726.17	208.84	36.35
CTS response time (switch)	Concern group	34	696.89	673.37	208.94	35.83
	Comparison group	33	867.65	802.88	249.84	43.49
WHO5	Concern group	34	4.41	4.50	0.85	0.15
	Comparison group	33	4.28	4.40	0.73	0.13
Frequency of experienced Gaming Addiction Scale (GAS) indicators	Concern group	34	2.76	2.50	0.91	0.16
	Comparison group	33	1.99	2.00	0.51	0.09

**FIGURE 3:**
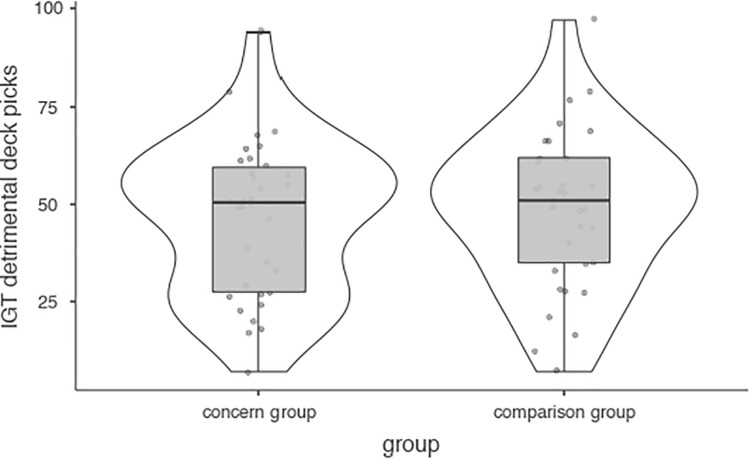
Iowa Gambling Task detrimental deck picks.

Participants chose the detrimental Iowa Gambling Task decks on average of 47.42 (*SD* = 19.97) out of 100 picks or just below chance level with quite a bit of variance. No significant difference was found between case and control participants (Figure 3). Mediation analyses did not detect significant mediation by weekly game time (see Appendix).

**FIGURE 4:**
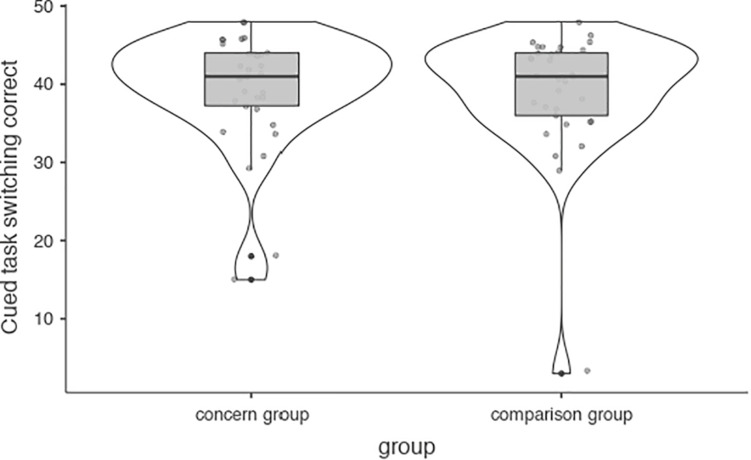
WHO5 general wellbeing.

Participants scored a mean of 39.18 (*SD* = 7.68) correct responses on Cued Task Switching, with mean response times of 709 ms (*SD* = 205.43) and 781 ms (*SD* = 243.90) for repeated and switched tasks, respectively. No significant difference was found between case and control participants, neither when requiring responses that fit congruently nor incongruently with the response required for the previous task (Figure 4). The case group, however, had significantly lower response times both for recurring tasks and after switches. Mediation analyses did not suggest that either of these differences were significantly mediated by weekly game time (see Appendix, bootstrapping was employed for the number of correct responses).

**FIGURE 5:**
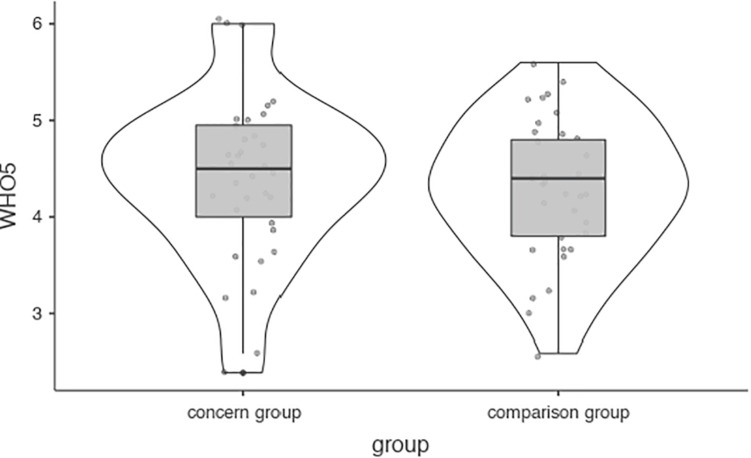
Cued task switching task.

Participants scored a mean of 4.34 (*SD* = 0.79) on the WHO5 measure of everyday wellbeing, suggesting general thriving in the sample. No significant difference was found between case and control participants (Figure 5). Mediation analyses did not detect significant mediation by weekly game time (see Table xx in the Appendix, bootstrapping was employed).

**FIGURE 6:**
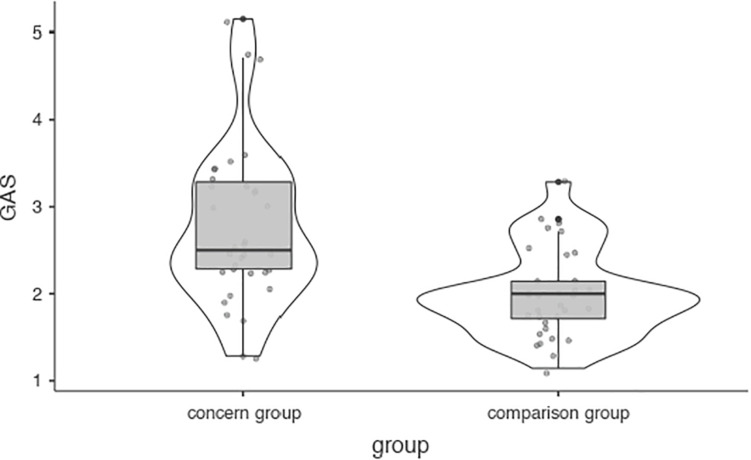
Gaming Addiction Scale.

Finally, participants scored a mean of 2.38 (*SD* = 0.83), falling between “rarely” and “sometimes,” when combining the seven experiences used in the Gaming Addiction Scale. Here, the case group scored significantly higher (“sometimes”) than controls (“rarely”) (Figure 6). The case group had a significantly higher number of individuals who would be considered addicted per GAS scores, χ^2^ (1,67) = 13.22 (*V* = 0.44, *p* > 0.001). Out of 34 individuals in the case group, 21 could be considered addicted (61.8%) in comparison with 6 out of 33 in the control group (18.2%) according to the most inclusive thresholds in the literature (Lemmens et al., 2011). Conversely, when using the more conservative threshold, in which individuals must respond with 4 or higher on each criteria, just 17.6% of the case group would be considered addicted, as opposed to 0% in the control group, which still constitutes a significant group difference, χ^2^ (1,67) = 6.40 (*V* = 0.31, *p* < 0.05).

We explored the relationships between dependent variables from the children’s survey and time spent playing. If game time is an issue, then that variable should predict our dependent variables. Weekly game time correlated with the frequency of experiences associated with the gaming addiction scale itself (*r* = 0.52, *p* < 0.001) but with neither of the cognitive measures or wellbeing (Table 4).

**TABLE 4:** Correlations between main variables.

	**Parent concern level**	**Weekly game time**	**Minutes gaming weekdays**	**Minutes gaming weekend**	**WHO5**	**IGT detrimental decks**	**CTS correct**	**GAS**
Concern level	–							
	–							
Weekly game time	0.35**	–						
	0.004	–						
Minutes_gaming weekdays	0.29*	0.96***	–					
	0.017	<0.001	–					
Minutes_gaming_weekend	0.39**	0.90***	0.74***	–				
	0.001	<0.001	<0.001	–				
WHO5	0.17	−0.28*	−0.31*	−0.18	–			
	0.167	0.023	0.011	0.146	–			
IGT detrimental decks	0.09	−0.05	0.00	−0.12	0.21	–		
	0.475	0.698	0.975	0.315	0.091	–		
CTS correct	0.09	0.12	0.11	0.12	−0.08	−0.09	–	
	0.470	0.323	0.363	0.343	0.509	0.469	–	
GAS	0.39**	0.52***	0.48***	0.50***	−0.31*	−0.03	−0.05	–
	0.001	<0.001	<0.001	<0.001	0.011	0.779	0.704	–
								

**FIGURE 7:**
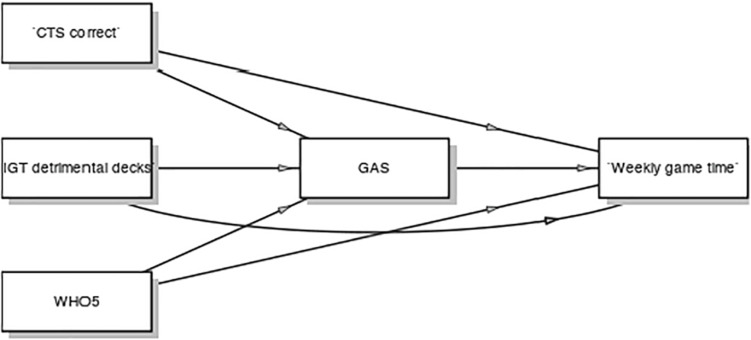
Model diagram.

Game Addiction Scale score was negatively related to the WHO5 score (*r* = −0.28, *p* = 0.02) but neither to CTS or IGT. Likewise, a GLM-based mediation analysis with parametric bootstrapping (Figure 7) revealed no direct relationships between game minutes per week and CTS or IGT, individually or combined. However, entering GAS as a potential mediator revealed an indirect negative relationship between WHO5 and weekly play time mediated by GAS score (Table 5). This suggests that spending time gaming *per se* was not negatively related to general wellbeing in the sample, but that gaming is related to wellbeing through “sometimes” experiencing gaming addiction indicators like conflicts with parents or facing the need to game less.

**TABLE 5:** Mediation analysis: indirect and total effects.

**Type**	**Effect**	**Estimate**	***SE***	**95% C.I. ^*a*^**		**β**	***z***	***p***
				**Lower**	**Upper**			
Indirect	CTS correct ⇒ GAS ⇒ weekly game time	−5.01	8.73	−22.98	11.23	−0.03	−0.57	0.57
	IGT detrimental decks ⇒ GAS ⇒ weekly game time	0.68	2.94	−5.17	6.35	0.01	0.23	0.82
	WHO5 ⇒ GAS ⇒ weekly game time	−217.59	102.22	−417.50	−16.82	−0.16	−2.13	0.03
Component	CTS correct ⇒ GAS	−0.01	0.01	−0.03	0.02	−0.07	−0.61	0.54
	GAS ⇒ weekly game time	654.31	133.06	382.63	904.22	0.49	4.92	<0.001
	IGT detrimental decks ⇒ GAS	0.00	0.00	−0.01	0.01	0.02	0.24	0.81
	WHO5 ⇒ GAS	−0.33	0.15	−0.62	−0.05	−0.32	−2.28	0.02
Direct	CTS correct ⇒ Weekly game time	19.58	12.30	−2.79	45.41	0.14	1.59	0.11
	IGT detrimental decks ⇒ weekly game time	0.29	7.75	−14.49	15.90	0.01	0.04	0.97
	WHO5 ⇒ weekly game time	−160.92	174.08	−486.03	196.36	−0.12	−0.92	0.36
Total	CTS correct ⇒ weekly game time	14.57	16.94	−18.63	47.77	0.10	0.86	0.39
	IGT detrimental decks ⇒ weekly game time	0.97	6.64	−12.05	13.99	0.02	0.15	0.88
	WHO5 ⇒ weekly game time	−378.51	167.49	−706.79	−50.23	−0.27	−2.26	0.02

^a^Confidence intervals computed with method: parametric bootstrap. *p < 0.05, **p < 0.01, ***p < 0.001.

### Exploratory Analyses

To understand the case group of worried parents better, we explored the survey items detailing specific concerns. As seen in Table 6, the parents agreed with all the concerns suggested, including games stealing time, affecting wellbeing, and having a negative impact on social and cognitive abilities. Out of these, concerns over time for family and friends both correlated significantly with parents’ overall level of worry, as did concerns over social and cognitive skills. This gives us a sense of the factors that fuel worry and confirmed that our choices of measures matched popular conceptions among worried parents. As seen in Table 4, however, parents’ worry doid not correlated with any of the main measures in this study except for their children’s experiences of addiction related factors like high absorption or conflicts surrounding gaming and, unsurprisngly, their game time – especially diring the weekend, where parents are present to observe and discuss gaming habits.

**TABLE 6:** Case group parents’ worries about the effects of games.

	**School**	**Leisure activities**	**Social time**	**Family time**	**Sleep**	**Mood and wellbeing**	**Social abilities**	**Ability to resist easy rewards**	**Ability to focus**
*N*	50	52	52	52	52	52	52	52	52
Mean	3.82	4.38	4.50	4.75	3.96	4.31	3.65	3.96	3.83
**Correlation with overall worry**									
*R*	0.17	0.17	0.34*	0.39**	0.05	0.31*	0.47***	0.53***	0.40**
*P*	0.24	0.23	0.01	0.01	0.71	0.24	<0.001	<0.001	0.01

*p < 0.05, **p < 0.01, ***p < 0.001.

To determine sources of the medium-sized negative relationship between GAS score and wellbeing observed above, we explored the relationship between parental worry and individual items constituting the WHO5 and GAS scales in the children’s survey. Simple correlation analyses (Table 7) revealed that parents’ worry correlated with GAS indicators related to their children’s degree of psychological and behavioral engagement with gaming, as well as experiences of outer *conflicts with others* including *attempts by others to limit gaming* sessions. Parental worry only correlated negatively with the children’s WHO5 item *of feeling relaxed*.

**TABLE 7:** Correlations of WHO5 items.

	**Parent concern level**	**GAS**	**WHO5 1 good mood**	**WHO5 2 relaxed**	**WHO5 3 energy**	**WHO5 4 rested**	**WHO5 5 meaning**
Concern level	–						
	–						
GAS	0.39**	–					
	0.001	–					
WHO5 1 good mood	−0.04	−0.20	–				
	0.77	0.11	–				
WHO5 2 relaxed	0.29*	−0.10	0.41***	–			
	0.02	0.41	<0.001	–			
WHO5 3 energy	0.05	−0.27*	0.34**	0.20	–		
	0.68	0.03	0.01	0.11	–		
WHO5 4 rested	0.15	−0.26*	0.20	0.17	0.41***	–	
	0.24	0.03	0.11	0.177	<0.001	–	
WHO5 5 meaning	0.11	−0.19	0.39**	0.43***	0.27*	0.43***	–
	0.38	0.13	0.001	<0.001	0.026	<0.001	–

*p < 0.05, **p < 0.01, ***p < 0.001.

In the children, the relationship between GAS and WHO5 (Table 8) appears to largely come from WHO5 items related to *feeling rested after sleep* and *energized during the day*, suggetsing that some ill-being from too much gaming could be related to lack of rest and sleep.

**TABLE 8:** Correlations of GAS items.

	**Parent concern level**	**WHO5**	**GAS01 constant thinking**	**GAS2 game time increasing**	**GAS3 playing to forget**	**GAS4 others attempt to limit**	**GAS5 feel bad if you cannot play**	**GAS6 conflicts with others**	**GAS7 neglecting other activities**
Concern level	–								
	–								
WHO5	0.17	–							
	0.17	–							
GAS 1 constant thinking	0.26*	−0.23	–						
	0.03	0.06	–						
GAS 2 more and more time	0.41***	−0.11	0.56***	–					
	<0.001	0.35	<0.001	–					
GAS 3 playing to forget	−0.08	−0.38**	0.10	−0.01	–				
	0.53	0.01	0.41	0.95	–				
GAS 4 others attempt to limit	0.37**	−0.07	0.33**	0.45***	0.18	–			
	0.01	0.59	0.01	<0.001	0.14	–			
GAS 5 feel bad if you cannot play	0.46***	−0.21	0.55***	0.67***	0.13	0.54***	–		
	<0.001	0.09	<0.001	<0.001	0.30	<0.001	–		
GAS 6 conflicts with others	0.43***	−0.10	0.54***	0.67***	−0.00	0.60***	0.71***	–	
	<0.001	0.44	<0.001	<0.001	0.99	<0.001	<0.001	–	
GAS 7 neglecting other activities	0.03	−0.38**	0.38**	0.32**	0.24*	0.30*	0.38**	0.39**	–
	0.79	0.01	0.002	0.01	0.055	0.01	0.001	0.001	–

*p < 0.05; **p < 0.01; ***p < 0.001.

Conversely, WHO5 scores correlated negatively with the GAS indicators *playing to forget real-world problems* and *neglecting non-gaming activities*. This supports the idea that excessive gaming can displace activities in other arenas and can sometimes be interpreted as a psychologically attractive avoidance behavior, which might obscure other sources of distress.

## Discussion

We set out to compare children of especially worried parents to similar young adults, in order to test if this group suffered from detriments to wellbeing, cognitive control, and indicators theoretically related to addiction. Apart from more frequently experiencing problems associated with the Gaming Addiction survey, such as wanting to play more and conflicts with parents, the data revealed that the children of worried parents were just as happy and well-functioning as other adolescents in their age group.

Overall, this suggests that many worried parents are ill equipped to judge gaming as problematic in terms of wellbeing and functional impairments.

The main difference between the groups was, unsurprisingly, that children to parents who worried about gaming played significantly more hours, and “sometimes” (as per the wording of the average response) experienced issues that the Gaming Addiction Scale takes as indicators of problems, that non-gamers rarely encounter. Instead of finding at a sample of mentally troubled youth, we gain a sense of the kind of otherwise average children, who may get labeled as problem gamers by worried parents.

Mediation analyses suggest that time spent gaming has had little discernable impact on cognitive control or everyday wellbeing. There was, however, an interaction between game time and wellbeing that related to the experience of the gaming addiction symptoms. In other words, even though the case group on average experienced the same level of wellbeing as other kids their age, those with lower wellbeing scores appeared to also experience a higher proportion of gaming addiction indicators in their day to day lives. This especially seemed to come from lack of rest and energy.

Paradoxically, parental worry correlated with the WHO5 item of feeling relaxed. The more worried the parent, the less relaxed the child reported to be in their everyday life. We are tempted to suggest that having highly involved parents makes the life of avid gamers more stressful, but perhaps this instead hints at a conflict between the subjective relaxation children derive from digital entertainment (indeed, the on-screen participants told us, that they played especially as a way to unwind), the effects on fatigue of late-night gaming sessions, and the motives, priorities, and understandings held by their parents. Indeed, family factors are found to have a consistent relationship to issues of problematic gaming and Internet use (Nielsen et al., 2020).

We also found a negative association between children’s wellbeing and gaming to escape real-world problems, as well as the feeling of neglecting non-gaming activities. This seems in line with previous research suggesting problematic gaming behavior can be a response to stressful, pre-existing problems (Snodgrass et al., 2014; Prax, 2016). Our findings thus support the notion that reasons for obsessive gaming must be sought in a broader ecological understanding of children’s life worlds (Nielsen et al., 2020).

Critical researchers have previously pointed to how the addition of Gaming Disorder to IDC-11 may result in an over-estimation of non-pathological participation in, e.g., online gaming communities as an addiction, as well as a stigmatization of an activity that may not present the danger (Van Rooij et al., 2018). The current study supports this concern in the finding that, although our concern group did score somewhat higher on gaming addiction symptoms, this GAS score was not directly related to negative effects on cognition and wellbeing, or issues of cognitive control which have been known to predict other behavioral addictions. This draws the criterion validity of measures like GAS into question for identifying truly problematic cases. Given our findings, we surmise that overtly worried actions by parents actually *feed back into* the total GAS score by generating conflicts in the home, which will, in turn, lead to higher GAS scores on these criteria related to conflicts with others—not to mention adding stress in the home and souring parent–child relations.

Our results stand in contrast to previous research showing significant functional impairment and diminished psychosocial wellbeing in relation to excessive gaming (Lemmens et al., 2009; Billieux et al., 2017; Myrseth et al., 2017). Since our sample is based on identifying worried parents, rather than cutting across large populations of gamers, this result may present an important lesson on the potential problems that face, e.g., councilors when worried parents approach them for help. In such situations, professionals therefore need screening tools that are finely tuned to distinguish between non-pathological play and pathological behaviors with functional impairment (Colder Carras and Kardefelt-Winther, 2018). In light of the case group’s high scores on wellbeing, it seems unlikely that 61.8% or perhaps even 17.6% were pathologically addicted to video games. The average response was that controls “rarely” experience problem gaming indicators, and only “sometimes” for the case group, which does not convey a sense of constant struggles or functional impairment. This is supported by the Iowa Gambling Task which has previously been used as a measure of impaired decision making in individuals prone to addictions. Kids who gamed more were actually faster, if not more accurate, in their reactions to cognitive tests, but did not show the inability to defer gratification found in addicts (Bailey et al., 2013; Irvine et al., 2013; Trotzke et al., 2019).

The correlation between frequency of GAS indicators and general wellbeing in the group as a whole could be taken as a sign of poor criterion validity for the addiction measure, if the problems did not actually come from functional impairment. As such, it may be that measures like GAS are able to detect nuisances and conflicts in the lives of otherwise well-functioning families, meaning that the, perhaps over-sensitive, conflict-related criteria could lead to a danger of false positives. Indeed, while research shows that negative correlations to, e.g., wellbeing and avoidant behaviors must first and foremost be found in the extreme ends of media use bell curves (Przybylski and Weinstein, 2017; Vannucci and Ohannessian, 2019), the lack of between-group difference in present study illustrates that it may be very hard for parents to assess “how much is too much.”

It must of course also be considered that this study was conducted for television. Data collection was conducted in collaboration with a national Danish TV station but with the research design at the full discretion of investigators through dialog with the journalist about the main concerns Danish parents might find interesting. The station covered the data collection process and initial research findings through the eyes of four on-screen families. Although much larger than formats where one or a handful of on-screen participants are used for “studies” on television, we still only achieved a relatively small sample compared with proper research studies. There is also a good chance that, even though most participants did not appear on screen, the relationship to a known television station could introduce biases in recruitment or responses. Getting a very worried sample of parents to participate was, however, part of the point for this study: We wanted only parents who were concerned enough to respond to the media and online recruitment messages and involve their child in the process. This study should thus not be taken to be representative of parents or gamers in general, but it would be a shame to let the data go to waste, instead of drawing back the curtain of our “made for TV” study. The strenghts of this study are also its weaknesses.

### Limitations

The sample of concerned parents was relatively small and based on media recruitment. While the case group was by no means representative, or amenable to recruitment based on *a priori* power analysis, results of research staged for TV rarely gets submitted to peer review, which makes it difficult for anyone to interpret their methods and results. Here, we present the preregistered procedure to ensure transparency of our work with the media and communication at the academic and popular levels alike. Furthermore, the broad media platform allowed for recruitment of highly concerned parents from across the country—a unique dataset which it would be a shame not to utilize fully. However, this recruitment opportunity also represents certain challenges. Working with parent–child dyads through online reporting runs a double risk of dropout—both for parents and children. However, once parents had consented and participated in their part of the study, most children followed suit. The greater concern might thus be to what extent the initiative of parents exacted demand characteristics on their children, even if the materials explicitly instructed them to leave their child to take part in the study alone. A few participants also disregarded the eligibility criteria and reported that they were not, in reality, very concerned. These families were eliminated from the case sample along with a number of potential participants who did not read far enough to accept the data policy and ethics instructions, suggesting that many were curious but either concerned about the nature of the study, or not motivated enough to fully participate as a parent–child dyad.

Furthermore, the technique of asking parents to fill in a survey, and subsequently pass the computer to their child, posed certain challenges. For instance, the child will likely have responded within the mental frame of existing discussions about gaming with their parents, which may have influenced the picture they paint of gaming. As described, we also lost quite a bit of data in the switches between parents and kids. Some control group kids failed to involve their parents beyond getting permission to participate, and some children in both groups responded without a code identifying the parents’ level of worry. Also, in order to retain full anonymity, the codes only conveyed the parent’s level of worry and the experimental group they were assigned to, so although it might have been interesting to couple more details about parents to their children’s responses, we opted not to create such a link.

This study also has the same shortfalls as other single dives into the complex lives of adolescents and their families. In the light of newer longitudinal research, long-term associations between media use and wellbeing seem tenuous (e.g., Jensen et al., 2019). As such, it is difficult to judge the extent to which our snapshot of young people’s lives, media behaviors, and wellbeing will mean much in 1, 5, or 10 years.

The relevance of the measures used can also be discussed. As discussed above, the GAS measure appears to have conceptual flaws, perhaps along with issues of sensitivity and precision. Other investigations have found prevalences of gaming addiction in adolescents and children ranging from 0.2% (Festl et al., 2013), 1.6% (Müller et al., 2015; Rehbein et al., 2015) to 4.6% (Fam, 2018). We are thus operating in a field, where criteria for addiction are not clearly established (Van Rooij et al., 2018). A major part criticism directed toward the gaming addiction diagnosis revolves around the lack of thorough, in-depth investigation of clinical symptoms rooted in exploration of self-identified problematic gamers, who are often children, rather than departing in diagnoses of existing addictions such as substance abuse in adults. As such, while GAS criteria may indicate problematic use, it is not well established whether the criteria accurately and comprehensively encapsulate the most relevant criteria of gaming *addiction*. In the current study, a negative relationship between game time and wellbeing was visible only if GAS symptoms were used as a statistical mediator, but in absolute terms, the case group had the same wellbeing scores as other kids their own age. This underscores how tick-a-box screening tools should never stand alone when making important decisions, particularly in a field as contested as gaming addiction.

This supports an alternative way of interpreting GAS scores, by dividing criteria into peripheral (indicative of high engagement) and core criteria taken to be indicative of severe problems suggested by some researchers in the field (Ferguson et al., 2011; Brunborg et al., 2015). In this approach, three items on GAS are peripheral while four are core criteria of gaming addiction. This approach significantly nuances the view that all problems and conflicts related to gaming should be taken as a sign of pathology, while still respecting that non-pathological nuisances and struggles are very real in many families.

Furthermore, even though the relationship between IGT and behavioral addiction is fairly well established, some studies contest this relationship, even finding higher IGT scores for non-pathological gamers compared with controls (Metcalf and Pammer, 2014). In addition, recent research suggests that cues related to a disordered behavior interferes with decision making in IGT (Trotzke et al., 2019), suggesting that problem gamers may be worse off at making decisions only when gaming is strongly on their mind—e.g., during play or when invited to play by friends. This perhaps limits the relevance of IGT as a test of negative cognitive effects or of resultant tendencies toward addictive behaviors, in the case group.

Finally, a few amendments to the analysis plan were needed after the initial preregistration. These changes are tracked at osf.io/hwbv4.

### Implications and Future Directions

Despite instances of worrying GAS scores in the present cohort, the study suggests that parents, who find themselves concerned over their child’s changes toward obsessive gaming, should not assume that their child is worse off than kids with other interests, or think of their child’s gaming in terms of pathology.

The commonalities between concern and control groups suggest that parents and even councilors should refrain from casually diagnosing children as “addicts,” and from assuming that gaming behavior is necessarily a source of detriments to wellbeing or cognitive functioning.

In cases where gaming feels like an increasing and persistent issue, parents and councilors should, it seems, pay attention to other potential sources of problems in the home, school, or peer group, while also helping the still immature child to make time for both rest/sleep and other activities which they might down-prioritize due to their gaming interests. As increased parental worry may feed into conflicts surrounding gaming behavior, which may in turn affect the relationship between problem gaming and wellbeing as our correlational analyses suggest. As such parents may, paradoxically, help their child more by worrying less—at least overtly.

A finding that warrants deeper scrutiny is the correlation between parental worry and gaming addiction indicators related to conflicts with others over gaming and others trying to limit your game time. These are likely very common experiences in any parent–child relationship. As such, the understandable and expectable behaviors of worried parents seem to be the source of at least part of the GAS diagnostic framework. In other words, we could be looking at a circular relationship: When a health professional uses an instrument like GAS to characterize the child of a worried parent as addicted, the frequency of experienced indicators could stem at least partially from the parents’ ensuing attempts at managing her media uses. In-depth studies of family experiences, conflicts, stressors, and negotiations about proper game time could be a key to understanding this paradox in depth, and perhaps to determine the appropriateness of scale items related to family conflict as diagnostic criteria for an addictive disorder in children.

Adolescence is never an easy time for anyone involved, fraught with changes and conflicts as it is. Gaming can definitely be problematic in a lot of ways, lead to practical and social conflicts, and take up enormous amounts of time, but at least in our small sample, children of worried parents were just as happy and cognitively healthy as other kids their age.

## Data Availability Statement

Pre-registration and materials available at the Open Science Framework (OSF) https://osf.io/hwbv4/.

## Ethics Statement

The studies involving human participants were reviewed and approved by Aarhus University, COBElab ethics committee. Written informed consent to participate in this study was provided by the participants’ legal guardian/next of kin.

## Author Contributions

Both authors contributed to planning, executing, and writing study. AL designed stimulus materials and wrote 70% of the manuscript text.

## Conflict of Interest

The authors declare that the research was conducted in the absence of any commercial or financial relationships that could be construed as a potential conflict of interest.
