# Supplementary material for: Can Worried Parents Predict Effects of Video Games on Their Children? A Case-Control Study of Cognitive Abilities, Addiction Indicators and Wellbeing
Source: Front Psychol. 2021 Jan 15;11:586699. doi: 10.3389/fpsyg.2020.586699 (PMC7848848; doi:10.3389/fpsyg.2020.586699)
Supplement: Supplementary file 1 [file Data_Sheet_1.docx]

Appendix: Mediation models

| Iowa Gambling Taksk: Indirect and Total Effects | | | | | | | | | | | | | | | | | | | | | |  |
| --- | --- | --- | --- | --- | --- | --- | --- | --- | --- | --- | --- | --- | --- | --- | --- | --- | --- | --- | --- | --- | --- | --- |
|  | | | | | | | | **95% C.I. (a)** | | | | |  | | | | | | | | |  |
| **Type** | | **Effect** | | **Estimate** | | **SE** | | **Lower** | | **Upper** | | | **β** | | | **Z** | | | **p** | | |  |
| Indirect |  | group ⇒ Weekly game time ⇒ IGT |  | .29 |  | 1.71 |  | -3.07 |  | | 3.64 |  | | .01 |  | | .17 |  | | .87 |  | |
| Component |  | group ⇒ Weekly game time |  | -630.79 |  | 108.89 |  | -844.22 |  | | -417.36 |  | | -.58 |  | | -5.79 |  | | < .001 |  | |
|  |  | Weekly game time ⇒ IGT |  | -.01 |  | .00 |  | -.01 |  | | .00 |  | | -.03 |  | | -.17 |  | | .87 |  | |
| Direct |  | group ⇒ IGT |  | .79 |  | 2.96 |  | -5.01 |  | | 6.60 |  | | .04 |  | | .27 |  | | .79 |  | |
| Total |  | group ⇒ IGT |  | 1.08 |  | 2.44 |  | -3.69 |  | | 5.86 |  | | .05 |  | | .44 |  | | .66 |  | |
| Note. (a) Confidence intervals computed with method: Standard (Delta method) | | | | | | | | | | | | | | | | | | | | | |  |
|  | | | | | | | | | | | | | | | | | | | | | |  |

| Cued Task Switching correct; Indirect and Total Effects (without bootstrapping) | | | | | | | | | | | | | | | | | | | | | |
| --- | --- | --- | --- | --- | --- | --- | --- | --- | --- | --- | --- | --- | --- | --- | --- | --- | --- | --- | --- | --- | --- |
|  | | | | | | | | **95% C.I. (a)** | | | | |  | | | | | | | | |
| **Type** | | **Effect** | | **Estimate** | | **SE** | | **Lower** | | **Upper** | | | **β** | | | **z** | | | **p** | | |
| Indirect |  | group ⇒ Weekly game time ⇒ CTS |  | -.72 |  | .00 |  | -.72 |  | -.72 |  | -.09 | |  | NaN | |  | NaN | |  |  |
| Component |  | group ⇒ Weekly game time |  | -630.79 |  | NaN |  | NaN |  | NaN |  | -.58 | |  | NaN | |  | NaN | |  |  |
|  |  | Weekly game time ⇒ CTS |  | .00 |  | NaN |  | NaN |  | NaN |  | 0.16 | |  | NaN | |  | NaN | |  |  |
| Direct |  | group ⇒ CTS |  | .55 |  | NaN |  | NaN |  | NaN |  | 0.07 | |  | NaN | |  | NaN | |  |  |
| Total |  | group ⇒ CTS |  | -.18 |  | .94 |  | -2.02 |  | 1.66 |  | -.02 | |  | -.19 | |  | .85 | |  |  |
| Note. (a) Confidence intervals computed with method: Standard (Delta method) | | | | | | | | | | | | | | | | | | | | | |
|  | | | | | | | | | | | | | | | | | | | | | |

| Cued Task Switching correct; Indirect and Total Effects (with bootstrapping) | | | | | | | | | | | | | | | | | | | | | |
| --- | --- | --- | --- | --- | --- | --- | --- | --- | --- | --- | --- | --- | --- | --- | --- | --- | --- | --- | --- | --- | --- |
| Indirect and Total Effects | | | | | | | | | | | | | | | | | | | | | |
|  | | | | | | | | **95% C.I. (a)** | | | | |  | | | | | | | | |
| **Type** | | **Effect** | | **Estimate** | | **SE** | | **Lower** | | **Upper** | | | **β** | | | **z** | | | **p** | | |
| Indirect |  | Group ⇒ Weekly game time ⇒ CTS |  | -.72 |  | .54 |  | -1.76 |  | | .36 |  | | -.09 |  | -1.34 |  | .18 | |  |  |
| Component |  | group ⇒ Weekly game time |  | -630.79 |  | 111.00 |  | -849.55 |  | | -414.46 |  | | -.58 |  | -5.68 |  | < .001 | |  |  |
|  |  | Weekly game time ⇒ CTS |  | .00 |  | .01 |  | -.01 |  | | .00 |  | | .16 |  | 1.38 |  | .17 | |  |  |
| Direct |  | group ⇒ CTS |  | .55 |  | 1.22 |  | -1.91 |  | | 2.86 |  | | .07 |  | .45 |  | .65 | |  |  |
| Total |  | group ⇒ CTS |  | -.18 |  | 0.94 |  | -2.02 |  | | 1.66 |  | | -.02 |  | -.19 |  | .85 | |  |  |
| Note. (a) Confidence intervals computed with method: Parametric bootstrap | | | | | | | | | | | | | | | | | | | | | |
|  | | | | | | | | | | | | | | | | | | | | | |

| Cued Task Switching: Response time after repeated task: Indirect and Total Effects | | | | | | | | | | | | | |
| --- | --- | --- | --- | --- | --- | --- | --- | --- | --- | --- | --- | --- | --- |
|  |  |  |  |  |  |  |  |  |  |  |  |  |  |
| **Type** | | **Effect** | | **Estimate** | | **SE** | | **β** | | **z** | | **p** | |
| Indirect |  | group ⇒ Weekly game time ⇒ CTS_repeat_RT_mean |  | -6.72 |  | 16.60 |  | -.03 |  | -0.40 |  | 0.67 |  |
| Component |  | group ⇒ Weekly game time |  | -630.79 |  | 108.89 |  | -.58 |  | -5.79 |  | < .001 |  |
|  |  | Weekly game time ⇒ CTS_repeat_RT_mean |  | .01 |  | .03 |  | .06 |  | .41 |  | .69 |  |
| Direct |  | group ⇒ CTS_repeat_RT_mean |  | 75.95 |  | 28.67 |  | .37 |  | 2.65 |  | .01 |  |
| Total |  | group ⇒ CTS_repeat_RT_mean |  | 69.23 |  | 23.61 |  | .34 |  | 2.93 |  | 003 |  |
| Note. (a) Confidence intervals computed with method: Standard (Delta method) | | | | | | | | | | | | | |
|  | | | | | | | | | | | | | |

| Cued Task Switching: Response time after switched task: Indirect and Total Effects | | | | | | | | | | | | | | |
| --- | --- | --- | --- | --- | --- | --- | --- | --- | --- | --- | --- | --- | --- | --- |
| Indirect and Total Effects | | | | | | | | | | | | | | |
|  |  |  |  |  |  |  |  |  |  |  |  |  |  |  |
| **Type** | | **Effect** | | **Estimate** | | **SE** | | **β** | | **z** | | **P** | | |
| Indirect |  | group ⇒ Weekly game time ⇒ CTS_switch_RT_mean |  | -15.28 |  | 19.68 |  | -.06 |  | -.78 |  | .44 |  |  |
| Component |  | group ⇒ Weekly game time |  | -630.79 |  | 108.89 |  | -.58 |  | -5.79 |  | < .001 |  |  |
|  |  | Weekly game time ⇒ CTS_switch_RT_mean |  | .02 |  | .03 |  | .11 |  | .78 |  | .43 |  |  |
| Direct |  | group ⇒ CTS_switch_RT_mean |  | 100.65 |  | 33.75 |  | .42 |  | 2.98 |  | .003 |  |  |
| Total |  | group ⇒ CTS_switch_RT_mean |  | 85.38 |  | 27.89 |  | .35 |  | 3.06 |  | .002 |  |  |
| Note. (a) Confidence intervals computed with method: Standard (Delta method) | | | | | | | | | | | | | | |
|  | | | | | | | | | | | | | | |

| WHO-5: Indirect and Total Effects | | | | | | | | | | | | | | | | | | | |  |
| --- | --- | --- | --- | --- | --- | --- | --- | --- | --- | --- | --- | --- | --- | --- | --- | --- | --- | --- | --- | --- |
|  | | | | | | | | **95% C.I. (a)** | | | |  | | | | | | | |  |
| **Type** | | **Effect** | | **Estimate** | | **SE** | | **Lower** | | **Upper** | | **β** | | **z** | | | **P** | | |  |
| Indirect |  | group1 ⇒ Weekly game time ⇒ WHO5 |  | .22 |  | .07 |  | 0.09 |  | .35 |  | .28 |  | | 3.38 |  | | < .001 |  | |
| Component |  | group1 ⇒ Weekly game time |  | -630.79 |  | 108.37 |  | -847.00 |  | -422.19 |  | -.58 |  | | -5.82 |  | | < .001 |  | |
|  |  | Weekly game time ⇒ WHO5 |  | -.01 |  | .01 |  | -.01 |  | -.01 |  | -.49 |  | | -3.93 |  | | < .001 |  | |
| Direct |  | group1 ⇒ WHO5 |  | -.28 |  | 10 |  | -.49 |  | -.08 |  | -.36 |  | | -2.73 |  | | .01 |  | |
| Total |  | group1 ⇒ WHO5 |  | -.06 |  | .10 |  | -.25 |  | .13 |  | -.08 |  | | -.66 |  | | .51 |  | |
| Note. (a) Confidence intervals computed with method: Parametric bootstrap | | | | | | | | | | | | | | | | | | | |  |
|  | | | | | | | | | | | | | | | | | | | |  |

|  |
| --- |
